# Durability and Improvement of Cement-Based Revetment Materials Serving in Subtidal, Intertidal, and Supratidal Environments

**DOI:** 10.3390/ma15093210

**Published:** 2022-04-29

**Authors:** Rui Sun, Dongmin Wang, Yiren Wang, Lei Zhang, Yue Gu

**Affiliations:** 1 School of Chemical and Environmental Engineering, China University of Mining & Technology-Beijing, Beijing 100083, China; sunrui_cumtb@163.com (R.S.); zhanglei3465@163.com (L.Z.); guyue1800@163.com (Y.G.); 2 Liaoning Yilifang Shaye Co., Ltd., Benxi 117000, China; 3 School of Environment and Civil Engineering, Dongguan University of Technology, Dongguan 523808, China; wangyr@dgut.edu.cn

**Keywords:** slag powder, polypropylene fibers, cement-based revetment materials, durability, chloride ion erosion, dry–wet cycles, deterioration characteristics

## Abstract

To improve the durability of cement-based revetment materials serving in different positions relative to the water level, slag powder and polypropylene fibers were added into cement to prepare paste, mortar, and concrete. Based on three simulated experiments of high-humidity air, dry–wet cycles-coupled chloride erosion, and complete immersion-coupled chloride erosion, the half-year durability of cement-based revetment materials was investigated. An abundant amount of Ettringite containing chloride was formed in the pores of the cement, and its formation was accelerated by dry–wet cycles. Replacing 30% of cement by slag powder and adding 0.1 vol.% of polypropylene fibers helped concrete in the intertidal zone to obtain a compressive strength of 47.58 MPa after erosion, equal to 159% of the reference. Slag powder was found to induce cement to form Friedel’s salt and C-S-H with a more amorphous structure, increasing its chemical binding ability and physical adsorption ability to chloride ions, and reduce the chloride ions’ penetration depth of concrete from 22.5 to 12.6 mm. Polypropylene fibers controlled the direction of surface cracks to be perpendicular to the specimen’s sides. These findings lay a foundation for the design of high-durability cement-based revetment materials serving in costal environments.

## 1. Introduction

Cement-based materials such as concrete have been widely used in urban construction under ordinary environments, due to the high availability of raw materials, mature construction technology, and excellent properties. However, the durability of cement-based materials serving in severe environments faces many challenges, such as abrasion, carbonization, freeze–thaw, and ion erosion [1,2,3]. The mechanical properties of cement-based materials mainly depend on the internal composition and structure, so the change of mechanical properties with time is regarded as the main essence of durability. Under the severe environment, the deterioration caused by external conditions would be accelerated when the composition deteriorates and the structure disintegrates, resulting in a rapid decline in mechanical properties [4,5]. According to Hou’s research [6], the total cost of corrosion damage in China in 2014 was 2127.82 billion yuan, accounting for 3.34% of China’s GDP in that year. Therefore, it is of great significance to investigate the deterioration mechanism of cement-based materials in severe environments, and to improve their durability.

The revetment infrastructure is built to defend against the water loss and soil erosion near the river bank or the coast. The protection zone can be divided into the subtidal zone, intertidal zone, and supratidal zone. Cement-based revetment materials (CBRM) are basic construction materials, widely used in hard revetment engineering. As a typical kind of CBRM, concrete for revetment faces completely different environmental challenges in different zones [7]. First, the subtidal zone is immersed in water forever, and the concrete serving in this zone suffers from chemical corrosion, such as sulfate ions and chloride ions, in addition to surf beat [8,9]. Second, the range of the intertidal zone is determined by the waterline between rising tide and falling tide. Although this range is periodically varied to the movement of the celestial bodies around the earth, most of the concrete serving here is in the long-term dry–wet cycles and chloride ion erosion coupling conditions. Third, the supratidal zone is located on the upper surface of the bank slope, and the evaporation of the water body causes the area to often be in a high-humidity air environment. For one thing, China’s coastline is 32,000 km-long and is increasing year-by-year, which has been gaining more and more attention in the area of soil and water conservation. For another, many inland rivers, lakes, and even wetlands also require huge investment in hard revetment [10,11]. The durability problems of concrete for revetment make it urgent for researchers to study its deterioration features and relevant improvement measures.

The deterioration of cement-based materials usually occurs under the combined action of physics and chemistry. In order to research the durability of CBRM in its service environment, it is necessary to trace its structure and composition evolution. Under the condition of dry–wet cycles, the chloride ion concentration inside the concrete increases due to water loss during the drying stage, thereby increasing the chloride ion concentration gradient between the invaded area and the non-invaded area, and further accelerating the penetration of chloride ions to deeper areas. The damage to concrete caused by chloride ions is mainly reflected in three aspects, as follows. First, chloride ions induce calcium ions to dissociate from the hydration product C-S-H gels and lead to a decrease in strength [12]. Second, the chloride ions crystallize with other cations to form crystals in the pore solution, and the induced expansion pressure leads to local structural damage [13,14]. Third, if steel bars are included in the concrete, chloride ions can reduce the pH value around the steel bars, thereby destroying the passivation film cover on the steel bar, and eventually leading to steel corrosion. The harm of stress concentration caused by steel corrosion is far greater than the crystallization pressure, which seriously damages the mechanical properties of CBRM [15].

In terms of improving the resistance to chloride ion corrosion of CBRM, most researchers use complete immersion as the corrosion condition to study the chloride ion transmission model under steady state [16,17,18]. Previous research shows that using mineral admixtures to replace part of the cement can optimize the pore structure of cement-based materials and improve the impermeability [19,20,21,22,23]. Otieno et al. [24] compared the effects of three different types of slag powder on the chloride ion conductivity of concrete, and the results showed that at a fixed water-to-binder ratio (W/B ratio), regardless of the type of slag used, chloride ions’ conductivity and porosity decreased with the increasing slag replacement level, affording better chloride penetration resistance. In addition, the study also found that Friedel’s salt (C_3_A·CaCl_2_·10H_2_O.) or Kuzel’s salt ((C_3_A·0.5CaCl_2_)·(0.5CaSO_4_)·11H_2_O) is the representative reaction product of cement-based materials exposed to a chloride ion attack. The reaction between chloride ions and aluminates rich in fly ash and slag powder can promote the formation of these two salts, thereby fixing chloride ions and improving the resistance to chloride ion penetration [25,26]. Sun’s study [27] further showed that the interlayer spacing of Friedel’s salt of cement-based materials will be reduced with the addition of fly ash and slag powder. Fibers can inhibit crack development, and therefore effectively improve the durability of cement-based materials. In terms of corrosion resistance, some fibers themselves are difficult to be eroded by chloride ions and have an enhanced impermeability of cracked concrete [28]. The studies of Berrocal [28], Chen [29], and Qureshi [30] showed that adding coarse fibers such as steel fibers can alleviate the surface deterioration of concrete under the coupled corrosion of dry–wet cycles and chloride ions. The influence of fine fibers against chloride ion penetration is affected by factors such as their length, content, and length–diameter ratio [29,31]. Polypropylene fibers, polyvinyl alcohol fibers, and basalt fibers are commonly used fine fibers, with single-fiber diameters below 200 μm. The amounts of fibers mixed into cement-based materials are usually calculated by volume fraction: for steel fibers, the amount is generally 0.2~0.8%, and the amount of fine fibers is generally 0.05~0.50% [32,33,34].

In this research, artificial simulation was carried out to investigate the durability of CBRM in supratidal, intertidal, and subtidal environments. A comparative study was performed between the CBRM using cement as cementitious component and the CBRM using cement blended with slag powder and polypropylene fibers. Different types of CBRM were concerned, including CBRM-Paste, CBRM-Mortar, and CBRM-Concrete. The deterioration characteristics of CBRM and its improvement by slag powder and polypropylene fibers under the intertidal environment were mainly explored by multi-measurements, involving the mechanical property test, chloride tracking, X-ray diffraction (XRD), and scanning electron microscope (SEM).

## 2. Materials and Methods

### 2.1. Raw Materials

**Figure 1:**
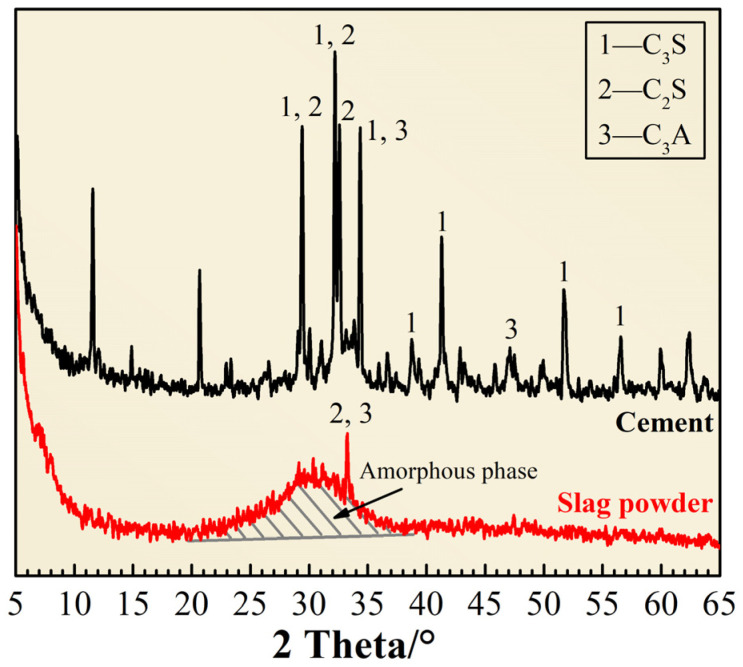
The mineral phase composition of cement and slag powder.

The cement used in this study was the diamond brand P.O 42.5R cement (C) produced by Hebei Yanxin Building Materials Group, with a specific surface area of 365 m^2^/kg and a density of 3.03 g/cm^3^. Slag powder (S) was made by grinding the granulated blast furnace slag discharged by Taiyuan Iron and Steel Group, with a density of 2.86 g/cm^3^ and a specific surface area of 501 m^2^/kg. The chemical compositions of cement and slag powder are shown in Table 1, and their mineral phase composition is shown in Figure 1. The polypropylene fibers (P) used here have a specific gravity of 0.91 g/cm^3^, a tensile strength of >458 MPa, and a length of 12 mm. The sand used for CBRM-Mortar was Chinese ISO standard sand. The sand used for CBRM-Concrete was quartz sand from Hebei, with a fineness modulus of 2.78 and good gradation (zone II, according to China standard GB/T 14685-2011). The crushing index was 5.6%. Polycarboxylic water-reducer (PCE) with a water-reducing rate of 30~35% was added to improve the workability of the CBRM-Concrete, CBRM-Paste, and CBRM-Mortar. The mixing water was tap water from Beijing. Sodium chloride (chemically pure) was used to prepare the immersion solution in order to simulate the hydrological environments.

### 2.2. Methods

#### 2.2.1. Specimen Preparation

**Figure 2:**
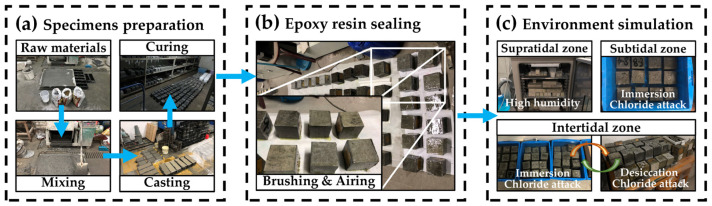
The preparation and service environment simulation of CBRM specimens.

CBRM-Paste, CBRM-Mortar, and CBRM-Concrete were prepared using raw materials according to the mixing ratios shown in Table 2, Table 3 and Table 4, respectively. All kinds of specimens were formed in the mold for 24 h and then demolded and kept under standard curing conditions (temperature at 20 ± 2 °C, RH ≥ 95%) for 28 days. CBRM-Concrete was taken as an example in Figure 2a to exhibit the process of specimen preparation. The specimens’ sizes of CBRM-Paste, CBRM-Mortar, and CBRM-Concrete were 30 × 30 × 30 mm, 40 × 40 × 160 mm, and 100 × 100 × 100 mm, respectively.

#### 2.2.2. Environment Simulation Procedure

(1) Simulation of the supratidal environment (high-humidity air): an environment with a humidity of 65% was provided by a humidified curing box to simulate the high-humidity air environment in the supratidal zone.

(2) Simulation of the intertidal environment (dry–wet cycles-coupled chloride ion erosion): Any side of the specimen (except the top and bottom surfaces formed when casting) was chosen to be the surface exposed to chloride. To ensure that the penetration of chloride ions in CBRM was one-dimensional during the simulation experiments, the other 5 surfaces were evenly painted with epoxy resin for sealing [35]. The dry–wet cycles experiment was carried out on the specimens after they were sealed and air-dried, and the immersion solution was a sodium chloride solution with a mass concentration of 5%. Each dry–wet cycle period was 5 days, which consisted of a procedure that involved immersing for 3 days and then drying for 2 days.

(3) Simulation of the subtidal environment (complete immersion-coupled chloride ion erosion): the specimens that had been sealed were immersed in a 5% sodium chloride solution for a long time.

The simulation experiments lasted for 180 days, and the immersion solution used for the intertidal zone and the subtidal zone was replaced every 30 days. Concrete was also taken as an example in Figure 2b,c to exhibit the process of epoxy resin sealing and environment simulation.

#### 2.2.3. Measurement Methods

**Figure 3:**
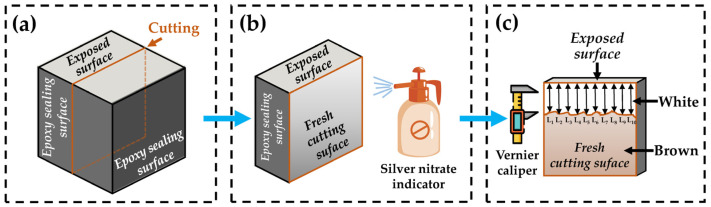
Measurement process of chloride ions’ penetration depth. (**a**) Specimen cutting, (**b**) indicator spraying, and (**c**) depth measurement.

**Figure 4:**
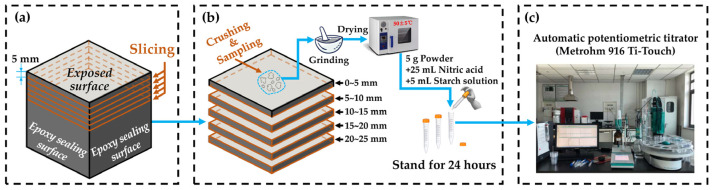
The measurement of chloride ions’ content at different depths. (**a**) Specimen slicing, (**b**) assay preparation, and (**c**) chloride ions’ content measurement.

From the date of specimen preparation, the macroscopic and microscopic properties were measured at 28, 28 + 30, 28 + 60, 28 + 90, and 28 + 180 days, respectively. The specific methods and conditions were as follows:

- Mechanical properties: the compressive strength and flexural strength of specimens were tested by an electronic universal testing machine.
- Macroscopic morphology of the exposed surface to chloride: Durability was visually analyzed by the macroscopic morphology comparison. A digital camera was used to take pictures of the exposed surface of the specimens serving in the intertidal zone and the subtidal zone.
- Chloride ions’ penetration depth: The chloride ion penetration depth was measured by the silver nitrate colorimetry. First, the specimen was cut into two halves along the direction perpendicular to the exposed surface. Second, the fresh cutting surface was immediately sprayed by the silver nitrate indicator with a concentration of 0.1 mol/L. Third, the chloride ion penetration area which was in white was traced by a waterproof pen, 15 min after spraying. Fourth, the depth from the exposed surface to the deep contour line was measured with a Vernier caliper. For one specimen, depth data from ten points equally spaced horizontally were collected to obtain the average value as the chloride ions’ penetration depth. If there was exactly a stone located at the measured point, the data of this point were immediately removed. The measurement process is shown in Figure 3.
- Chloride ions’ profile: As shown in Figure 4, the part of the specimen near the exposed surface was sliced into 5 pieces with a thickness of 5 mm. Samples from each piece were taken for grinding and drying before chloride ions’ content measurement, and the sampling point should be avoided where there are coarse aggregates. Then, 5 g of the dried powder obtained above was added into 25 mL of nitric acid solution (prepared with nitric acid and distilled water in a volume ratio of 1:7) and 5 mL of starch solution with a concentration of 10 g/L. Standing for 24 h would help the powder to fully dissolve. Then, chloride ions’ content was measured using a Metrohm 916 Ti-Touch automatic potentiometric titrator.
- Composition, microstructure, and morphology of hydration products: CBRM-Paste after 28 + 180 days of the simulation experiment was used to investigate the composition, microstructure, and morphology of hydration products under the chloride ion erosion. Powder samples and thin-sliced samples taken from the 0~5 mm zone of CBRM-Paste were dried in the same drying procedure as in Chloride ions’ profile test. Powder samples were scanned with a Bruker D8 Advance X-ray diffractometer (XRD) to explore the composition of the hydration products. The microstructure and morphology of thin-sliced samples with a thickness of 3~5 mm were observed using a JEOL JSM-6700F and a Phenom Pharos G1 scanning electron microscope (SEM).

## 3. Results and Discussion

### 3.1. Durability of CBRM Serving in a Supratidal Environment

#### 3.1.1. Mechanical Properties

**Figure 5:**
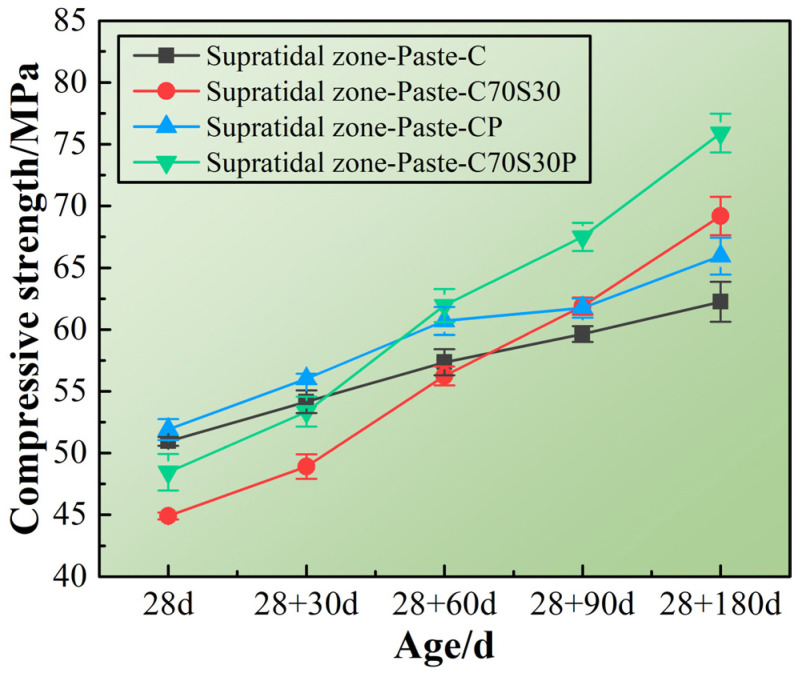
The mechanical properties of CBRM-Pastes serving in the supratidal zone simulated environment.

Figure 5 shows the compressive strength of CBRM-Paste serving in the simulated environment of the supratidal zone. On one hand, high humidity inhibited the evaporation of water inside the paste at early ages, thereby preventing the formation of internal pores. On the other hand, external moisture can also diffuse into the paste at late ages, contributing to the hydration of cement and slag powder. These two effects made the compressive strength gradually increase [36,37]. Among the four specimens, the compressive strength of Paste-C prepared with pure cement reached 50.95 MPa after standard curing for 28 days, and then gradually developed to 62.25 MPa after simulated service for 180 days. For Paste-C70S30, its compressive strength was lower than that of Paste-C in the first 28 + 60 days, and then gradually surpassed Paste-C and reached 69.20 MPa at 28 + 180 days. This is because the pozzolanic reaction of slag powder carried out in the alkaline environment produced by cement hydration resulted in a lower hydration degree before 28 + 60 days, but gradually increased to higher in the later period.

The improvement of the mechanical properties by adding polypropylene fibers is also shown in Figure 5. Paste-CP and Paste-C70S30P represent the cement paste and cement-slag powder composite paste mixed with polypropylene fibers, respectively. Polypropylene fibers’ reinforcement effect lasted from early ages to the later ages. Compared with Paste-C, the compressive strength of Paste-CP at 28 + 180 days increased by 5.94%; Compared with Paste-C70S30, the compressive strength of Paste-CP at 28 + 180 days increased by 9.68%. Polypropylene fibers helped Paste-C70S30P to obtain a compressive strength close to that of Paste-CP at 28 + 30 days, avoiding the low early strength caused by adding slag powder. The results of the above mechanical properties’ investigation showed that the high-humidity environment in the supratidal zone is conducive to the slow enhancement of the strength of CBRM. The addition of slag powder can significantly increase the later strength, and the simultaneous addition of slag powder and polypropylene fibers can further improve the mechanical properties at all ages.

#### 3.1.2. Hydration Products

**Figure 6:**
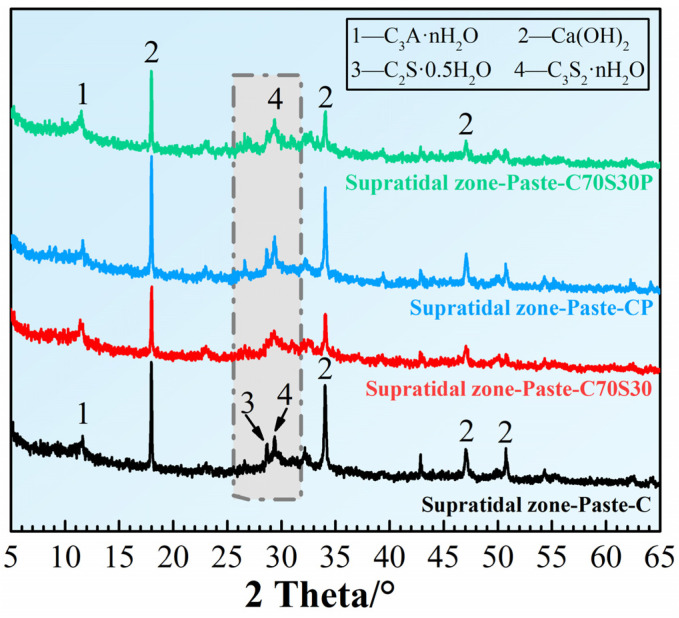
The XRD pattern of CBRM-Pastes servicing in the supratidal zone simulated environment at 28 + 180 days.

The hydration products of the four CBRM-Pastes serving in the supratidal zone for 180 days were investigated. The results from the XRD patterns (Figure 6) showed that the main hydration products included Ca(OH)_2_ with high crystallinity, calcium aluminate hydrates (C-A-H), and calcium silicate hydrates (C-S-H). C-A-H and C-S-H make major contributions to the mechanical properties of cement [38,39]. For Paste-C, a weak diffraction peak of C_3_A·H_2_O was observed around 11.6°, but the diffraction peaks of the two kinds of C-S-H (C_2_S·0.5H_2_O and C_3_S_2_·nH_2_O) that responded in the range of 25~32° were relatively sharp, indicating that they have a certain ordered crystal structure. For the Paste-C70S30, firstly, the pozzolanic reaction of slag powder led to the decrease of Ca(OH)_2_ content and the weakening of diffraction peak intensity; secondly, due to the more C_3_A and C_2_S supplied by slag powder, the diffraction peak intensity of C_3_A·nH_2_O was enhanced. The diffraction peaks of C_2_S·0.5H_2_O and C_3_S_2_·nH_2_O became weak when slag powder was added, proving that the slag powder promoted C-S-H formed with a more amorphous structure. Polypropylene fibers also slightly promoted cement hydration to a certain extent, which manifested in the little enhancement of the diffraction peak intensity of the hydration products, which is consistent with the analysis performed above in terms of mechanical properties.

### 3.2. Durability of CBRM Serving in the Intertidal Environment

#### 3.2.1. Mechanical Properties

**Figure 7:**
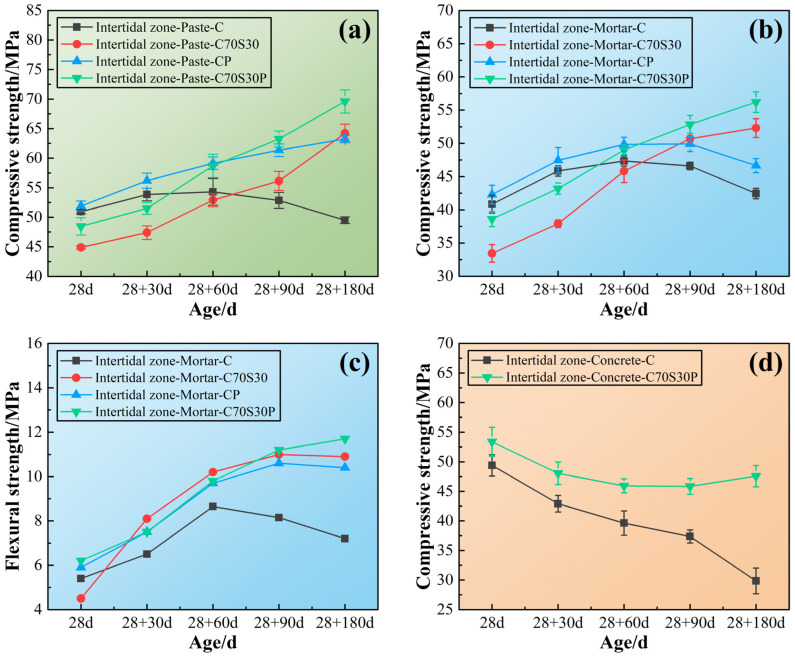
The mechanical properties of CBRM serving in the intertidal zone simulated environment. (**a**) CBRM-Paste, (**b**,**c**) CBRM-Mortar, and (**d**) CBRM-Concrete.

The mechanical properties of CBRM-Paste, CBRM-Mortar, and CBRM-Concrete serving in the intertidal zone were tested, and the results are shown in Figure 7. First, the durability differences among the paste, mortar, and concrete were compared. Take Paste-C, Mortar-C, and Concrete-C as examples, whose cementitious materials were made up of pure cement. The mechanical properties of both Paste-C and Mortar-C showed a trend of increasing at first and then decreasing under the action of long-term dry–wet cycles-coupled chloride ion erosion. The compressive strength Concrete-C decreased from the start of the intertidal zone simulation experiment, reducing to 29.85 MPa after 180 days of dry–wet cycles-coupled chloride ion erosion, which is only 60.43% of that before the simulation (at 28 days). Compared with Concrete-C, the mechanical properties of Paste-C and Mortar-C decayed after 60 days of dry–wet cycles-coupled chloride ion erosion, because the porosity of concrete is higher than that of paste and mortar.

Second, the influence of slag powder and polypropylene fibers on the durability of CBRM in this environment is discussed. For polypropylene fibers, similar to the situation in the supratidal environment, the addition of polypropylene fibers improved the mechanical properties. The compressive strength of Paste-CP was 63.25 MPa at 28 + 180 days, which is 127.78% of Paste-C. As the increase by polypropylene fibers in the supratidal zone was only 105.94%, a conclusion could be made that polypropylene fibers’ reinforcement to cement-based materials was not poorly influenced by dry–wet cycles-coupled chloride ion erosion. This may be due to the fact that the polypropylene fibers were dehydrated during the drying period of the drying–wetting cycles and maintained good toughness, thereby improving the strength [40]. The reinforcing effect of polypropylene fibers only comes from the reinforcement of the structure. Although the strength of CBRM with added polypropylene fibers was improved at all ages, it was still difficult for CBRM to avoid deterioration under long-term erosion. This was because polypropylene fibers as an organic additive did not fundamentally change the hydration reaction process of cement, nor did it change the type, structure, or properties of hydration products. For slag powder, the pozzolanic reaction of silicates from slag powder changed the process of cement hydration. Although the early strength of CBRM blended with slag powder was lower than that of the blank specimen, the C-S-H gels produced by pozzolanic reaction gradually filled the pores, providing a continuous strength increase to CBRM [41]. Taking concrete as an example, the interface transition zone is a weak area in the concrete with a loose structure and rich pores. The hydration products of slag powder gradually fed into the structure defectiveness of the interface transition zone at later ages, meaning the compressive strength of Concrete-C70S30P from 28 + 90 to 28 + 180 days did not decrease but increased (Figure 7d).

#### 3.2.2. Macroscopic Morphology of the Exposed Surface

**Figure 8:**
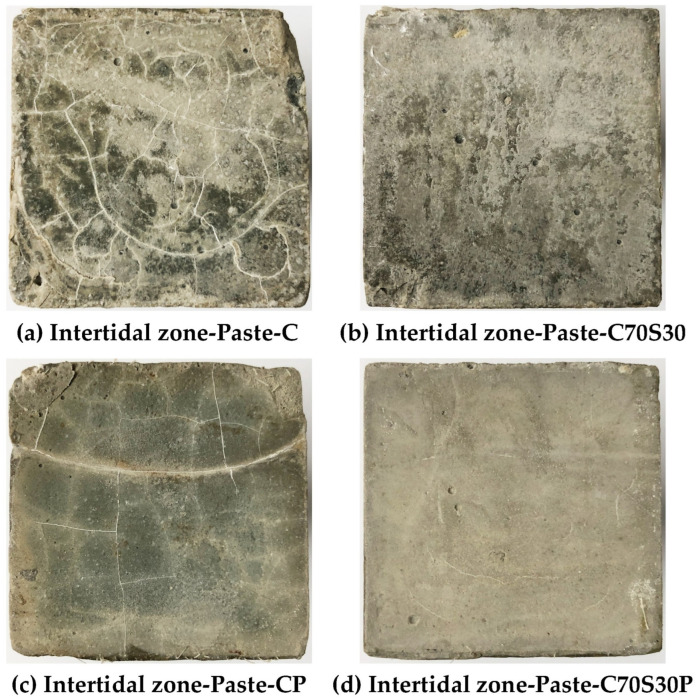
The macroscopic morphology of the exposed surface of CBRM after 180 days of dry–wet cycles-coupled chloride ion erosion.

The macroscopic morphology of the exposed surface of paste after 180 days of dry–wet cycles of the chloride ion attack was recorded and studied, as shown in Figure 8. The exposed surface integrity of the four CBRM-Pastes from good to bad is as follows: Paste-C70S30P > Paste-C70S30 > Paste-CP > Paste-C. The exposed surface of Paste-C (Figure 8a) showed many interlaced cracks under long-term erosion, and the gaps were filled with white sodium chloride crystals. Obvious exfoliation and other deterioration phenomena can be observed around the exposed surface. Chloride ion penetration is very sensitive to the relative humidity both inside and outside the specimens [42,43]. Sodium chloride solution penetrated into the exposed surface during the immersion stage, and then lost water and crystallized in the desiccation stage, bringing about severe crystallization pressure which led to the destruction of CBRM. Compared with the Paste-C, the addition of polypropylene fibers significantly inhibited the formation and development of cracks (Figure 8c). In terms of crack quantity, the number of cracks on the exposed surface of the Paste-CP was significantly less than that of the Paste-C. In terms of crack morphology, the cracking on Paste-CP was mainly along the direction approximately perpendicular or parallel to the four sides of the specimen. The crisscross cracks on Paste-CP indicated that its internal cracks were constrained by polypropylene fibers to avoid structural breakup. The cracks on the exposed surface of Paste-C mainly had a chaotic arc shape, having a high degree of mutual intersection, which also reflected the high crystallization pressure inside. Multiple cracking points were easily interfered with by nearby erosion and formed a crack network, increasing the risk of damage to Paste-C.

For Paste-C70S30 prepared by using slag powder to replace part of the cement (Figure 8b), although its exposed surface was covered by partially crystallized sodium chloride, no obvious cracks were observed. Paste-C70S30P with both slag powder and polypropylene fibers added (Figure 8d) exhibited the best smoothness degree of the exposed surface. The hydrophobicity of the polypropylene fibers reduced the penetration rate of chloride ions into the exposed surface and reduced the nucleation sites for NaCl or CaCl_2_ crystals during the desiccation stage. The physical adsorption and chemical bonding to chloride ions by C-S-H gels produced from slag powder can reduce the rate of chloride ion migration to deeper areas [19,20,44]. The blocking effect of slag powder on chloride ions will be proven by the hydration and erosion products analysis in Section 3.2.6.

#### 3.2.3. Internal Microstructure and Morphology

**Figure 9:**
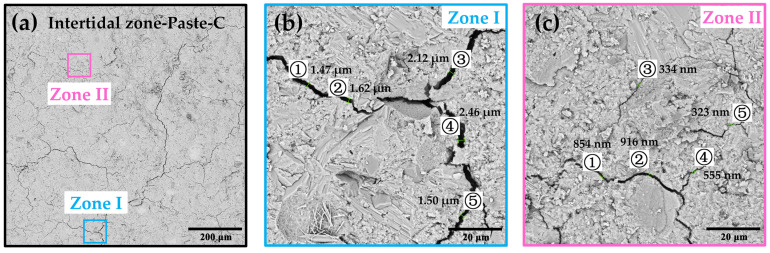
The SEM photos of internal cracks in Intertidal zone-Paste-C at (**a**) 300 magnification and (**b**,**c**) 3000 magnification.

On the basis of the macroscopic morphology of the exposed surface, the internal microstructure and morphology caused by the erosion were studied. Figure 9 shows the cracks found in Paste-C serving in the intertidal zone. The distribution of cracks was similar to that on the exposed surface. The width of cracks, measured at 10 positions in Figure 9b,c, ranged from hundreds of nanometers to 3 μm. The wide cracks became connected through the narrow cracks. Some crystals can be observed around the cracks, which may be the main reason for the cracks.

**Figure 10:**
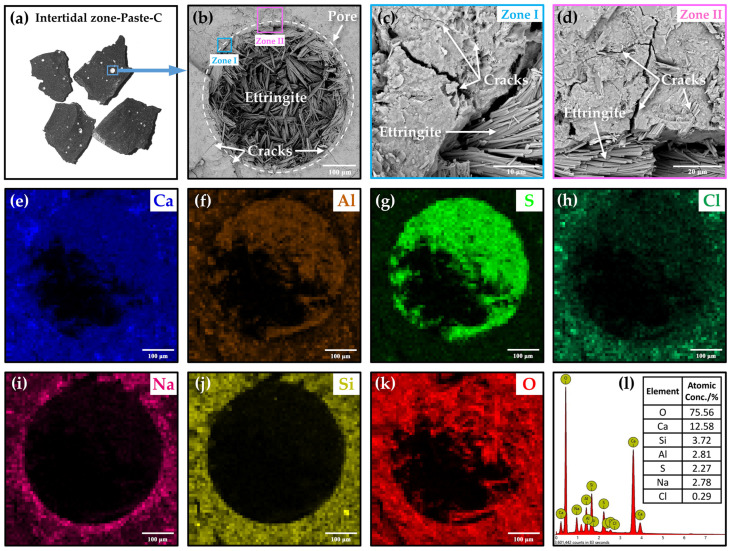
The internal microstructure deterioration characteristics of Paste-C after 180 days of dry–wet cycles-coupled chloride ion erosion. (**a**) The internal section photo of Intertidal zone-Paste-C. The SEM photos of internal microstructure at (**b**) 520 magnification, (**c**) 7200 magnification and (**d**) 3800 magnification. (**e**–**k**) The EDS-Mapping of (**b**). (**l**) The elements content of (**b**).

Figure 10 shows the internal microstructural and elements’ distribution of the Paste-C after 180 days of dry–wet cycles-coupled chloride ion erosion. Due to the influence of the fluidity when the paste was mixed, a small amount of bubbles formed in the fresh paste. The water in the bubbles gradually evaporated as paste hardened, and the bubbles developed into pores (Figure 10a). During the immersion stage of the dry–wet cycles, the immersion solution entered into the specimens under the action of osmotic pressure, re-wetting the pores. Water in pore solution evaporated again during the desiccation stage.

**Figure 11:**
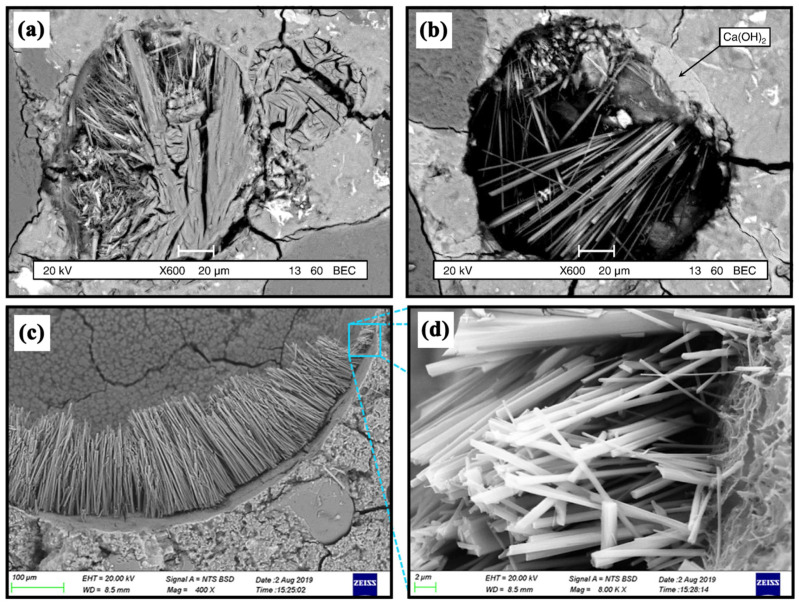
The secondary Ettringite found by Rosenqvist et al [45] and Neumann et al. [46]. (**a**,**b**) The microstructure of concrete after 55 years of exposure to river water. Reprinted/adapted with permission from Ref. [45]. 2017, Elsevier. (**c**,**d**). The microstructure of concrete after 40 years of exposure to river water. Reprinted/adapted with permission from Ref. [46]. 2021, Elsevier.

Ettringite (C_3_A·3CaSO_4_·32H_2_O) is an important hydration product that exists in the early age of cement. However, abundant needle-shaped crystals (Figure 10b) were observed in the pores of Paste-C after half a year of erosion. Many cracks (Figure 10c,d) which may be caused by these crystals were also found around the pore. Actually, these needle-shaped crystals were identified to be secondary Ettringite by EDS-mapping, shown in Figure 10e–l. There are three important characteristics in the EDS-mapping results. First, Ca, Al, S, and O elements, but no Si element, were obviously detected in the zone rich in the needle-shaped crystals, proving that they were Ettringite. Second, scarcely any Na could be detected in the pore (or on the Ettringite), but it was found to bind tightly to the pore boundaries. Third, Cl was detected not only outside the pore but also distributed in the Ettringite inside the pore. The above results were consistent with the findings by Rosenqvist et al. [45] and Neumann et al. [46], who studied the concrete after 55 and 40 years’ exposure to river water (Figure 11). However, the Na and Cl related to secondary Ettringite were first researched in this paper.

Therefore, it was deduced that Na^+^ and Cl^−^ in the immersion solution penetrated into the materials and arrived at the boundaries of pores. Only Cl^−^ promoted the formation of Ettringite and bonded to the needle-shaped crystals grown into the pores, but Na^+^ stopped transportation and gathered around the pores. The dry–wet cycles accelerated the penetration of Na^+^ and Cl^−^, and Cl^−^ further induced the massive formation of Ettringite, which eventually led to cracking. 

**Figure 12:**
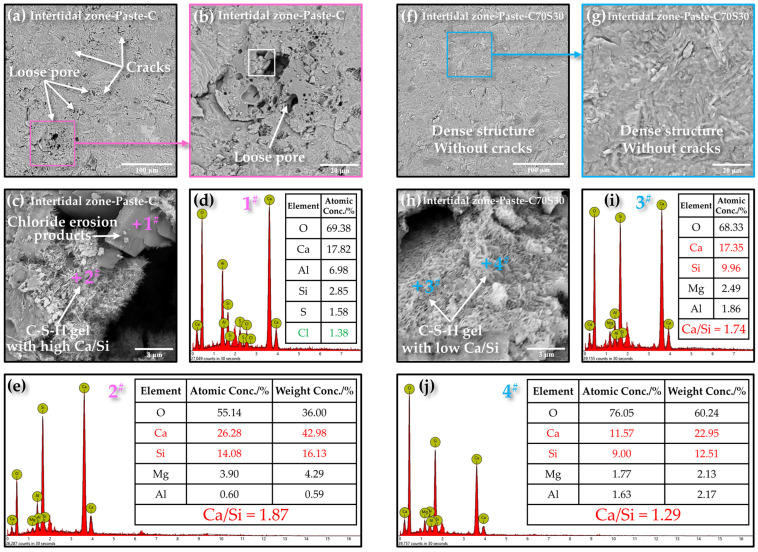
The influence of slag powder on the internal microstructure of CBRM-Paste after 180 days of dry–wet cycles-coupled chloride ion erosion. The SEM photos of Intertidal zone-Paste-C at (**a**) 800 magnification, (**b**) 3000 magnification, and (**c**) 20,000 magnification. The EDS results of spot 1^#^ (**d**) and 2^#^ (**e**). The SEM photos of Intertidal zone-Paste-C70S30 at (**f**) 800 magnification, (**g**) 3000 magnification, and (**h**) 20,000 magnification. The EDS results of spot 3^#^ (**i**) and 4^#^ (**j**).

The microstructure and morphology of Paste-C and Paste-C70S30 are compared in Figure 12 to investigate the influence of slag powder on the internal microstructure of CBRM after erosion. As shown in Figure 12a,b, in addition to cracks due to erosion, a large area with loose pore structures was also observed in the matrix. The rapid hydration of C_3_S in cement caused some cement particles to be wrapped by other hydration products and not hydrated at early ages, forming a shell-core structure. When the outer shell of this shell-core structure was damaged due to chlorides’ crystallization pressure, the non-hydrated particles inside with a loose structure would be exposed, and it will further accelerate the penetration of chloride ions to the interior. The addition of slag powder particles inside the cement hydrate shell not only delayed the hydration of cement, but also reacted with Ca(OH)_2_, making the shell-core structure gradually become a dense matrix (Figure 12f,g), increasing the ability of CBRM to resist chloride ion attacks. Figure 12c,h show the C-S-H gel in Paste-C and Paste-C70S30. The Ca/Si ratio of C-S-H in Paste-C70S30 (Ca/Si = 1.74 at spot 3#, Ca/Si = 1.29 at spot 4#) was lower than that in Paste-C (Ca/Si = 1.87 at spot 2#), and the low Ca/Si ratio contributed to the high strength [47]. The crystals containing Ca, Al, Si, and Cl were observed in Paste-C (Figure 12c), whose crystallization process is also one of the main causes of cracking.

**Figure 13:**
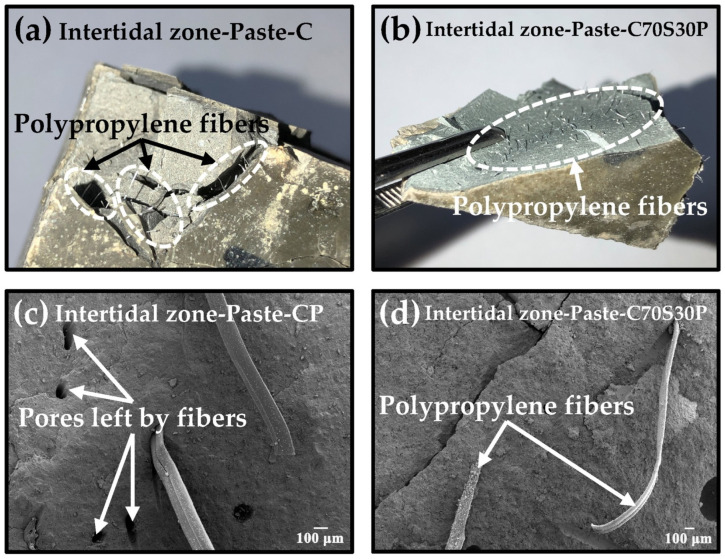
The influence of polypropylene fibers on the internal microstructure of CBRM-Paste after 180 days of dry–wet cycles-coupled chloride ion erosion.

Figure 13 shows the reinforcement effect of polypropylene fibers on CBRM-Paste on macroscopic and microscopic scales. Figure 13a,b are the macro photos of the fracture of the Paste-CP and Paste-C70S30P after the mechanical properties test, which had sustained 180 days of dry–wet cycles-coupled chloride ion erosion. It can be seen from the fracture surface that the polypropylene fibers were uniformly distributed in the paste. After the mechanical properties test, only a small number of pieces fell off the CBRM-Paste with added polypropylene fibers. The separated parts were still bridged with each other through the fibers, which ensured the integrity of the structure and the ability to suffer light loads. This is very important for revetment materials serving in the intertidal zone, which means that even if damaged at local sites, the presence of polypropylene fibers can help the revetment material continue to resist continuous water wave impact [48]. Figure 13c,d show the SEM photos of Paste-CP and Paste-C70S30P. Many pores were observed in the Paste-CP without adding slag powder, which were left by the removal of polypropylene fibers, showing the insufficient adhesion between the fibers and Paste-CP. Instead, pores left by polypropylene fibers were rarely found in the Paste-C70S30P. A conclusion can be made that slag powder can improve the adhesion between the polypropylene fibers and paste, forming a relatively dense interface structure [49].

#### 3.2.4. Chloride Ions’ Penetration Depth

**Figure 14:**
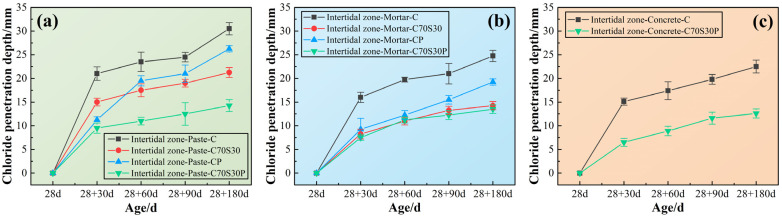
The chloride ions’ penetration depth of CBRM serving in the intertidal zone simulated environment. (**a**) CBRM-Paste, (**b**) CBRM-Mortar, and (**c**) CBRM-Concrete.

In order to reveal the relationship between macroscopic durability and the microscopic erosion process, the chloride ions’ penetration depth of CBRM-Paste, CBRM-Mortar, and CBRM-Concrete at different ages was studied. As shown in Figure 14, the penetration depth of the three types of materials in the same age are as follows: CBRM-Concrete > CBRM-Mortar > CBRM-Paste, which seems to be contrary to the viewpoint that “the more pores there are, the easier it is to deteriorate” proposed in Section 3.2.1. Although this phenomenon did not interfere with the analysis of the effect of slag powder and fibers on the durability of the same type of material, a reasonable speculation is provided here to explain the contradiction. The reason can be attributed to the interference of the aggregates on the chloride ions’ depth measured by the silver nitrate colorimetry. Chloride ions are considered to migrate and enrich only in the paste. First, the presence of sand and stone in concrete could block the direct penetration of chloride ions. Second, after spraying the silver nitrate indicator on the cutting surface, the boundary of the chromogenic area often intersects with the aggregates, causing confusion on the outline, which leads to the small measurement result.

For CBRM-Paste, polypropylene fibers could reduce the chloride ions’ penetration depth of both Paste-C and Paste-C70S30 at different ages. For CBRM-Mortar, the decreasing effect of polypropylene fibers on the chloride ions’ penetration depth of Mortar-C70S30 was far inferior to that in CBRM-Paste. As there are more pores in CBRM-Mortar than CBRM-Paste, the penetration of chloride ions was promoted by the pores rather than cut off by polypropylene fibers. Although the pozzolanic reaction of the slag powder was not carried out on a large scale in early ages, its ability to fix chloride ions was significantly improved, so that the chloride ions’ penetration depth was reduced from 21 mm of Paste-C to 15 mm of Paste-C70S30 at 28 + 30 days. For CBRM-Concrete, the chloride ions’ penetration depth of Concrete-C70S30P was only 12.6 mm at 28 + 180 days. Under the synergistic effect of slag powder and polypropylene fibers, the chloride ions’ penetration depth of Concrete-C70S30P at each age was always about half of that of Concrete-C.

#### 3.2.5. Chloride Ions’ Profile

**Figure 15:**
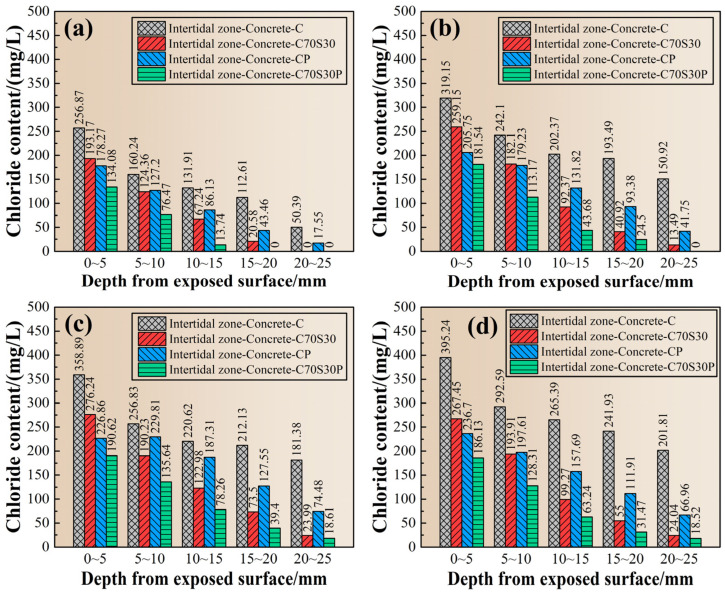
The chloride ions’ profile of CBRM-Concrete serving in the intertidal zone simulated environment. (**a**) 18 + 30 days, (**b**) 18 + 60 days, (**c**) 18 + 90 days, and (**d**) 18 + 180 days.

Since the chloride ions penetrated simultaneously with the hydration reaction of cement or slag powder, the penetration resistance of chloride ions increased with the increase of ages, and also increased with the distance away from the exposed surface. Since the lowest detection limit for chloride ions’ content by silver nitrate colorimetry was still too high, it make the chloride ions’ penetration depth measurement difficult to accurately reflect the erosion situation of chloride ions. In order to explore the erosion by chloride ions in different regions away from the exposed surface, the chloride ions’ content of CBRM-Concrete was detected from the regions at 0~5, 5~10, 10~15, 14~20, and 20~25 mm. The results are shown in Figure 15. First, the chloride ions’ content of the same specimen decreased with the depth at a fixed age. Secondly, the chloride ions’ content in each region increased with the age, and the chloride ions’ content in the region of 0~5 mm of Concrete-C reached 395.24 mg/L after 180 days of erosion.

Although both slag powder and polypropylene fibers enhanced the mechanical properties of CBRM in the intertidal zone, their inhibition effects on chloride ions were different. It can be found in Figure 15, at all ages, for the shallow region (0~5 mm), that the inhibition effect of polypropylene fibers on chloride ions’ penetration is better than that of slag powder. While in the deep region (depth > 5 mm), the inhibiting effect of slag powder was better than that of polypropylene fibers. 

The reason can be explained as follows: due to the hydrophobicity of polypropylene fibers, they tended to assemble around the sidewall of molds during casting, so the fiber content in the shallow region would be higher than that in the inner region, providing better resistant capability to chloride ions [49,50]. The C-S-H gels (Figure 12h) generated by the continuous hydration of slag powder refined the pores of CBRM, increasing its physical adsorption ability and chemical bonding ability to chloride ions. The deep region in cement-based materials, less vulnerable to carbonation, usually has a higher hydration degree than the outer region, and therefore the resistant capability to chloride ions of slag powder in the deep region becomes the dominant one.

#### 3.2.6. Hydration and Erosion Products

**Figure 16:**
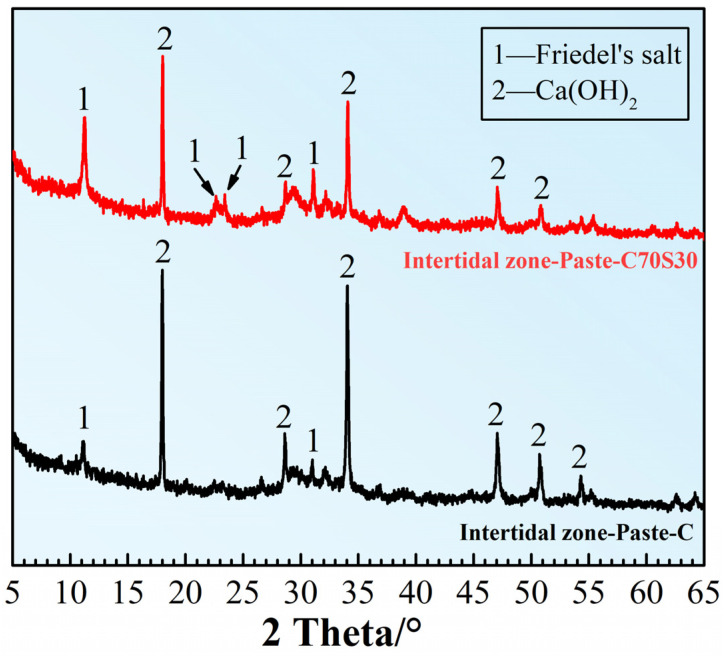
The influence of slag powder on the composition of CBRM-Paste after 180 days of dry–wet cycles-coupled chloride ion erosion.

The hydration and erosion products of Paste-C and Paste-C70S30 serving in the intertidal zone were studied after 180 days of dry–wet cycles-coupled chloride ion erosion. The XRD patterns of these two CBRM are shown in Figure 16. Friedel‘s salt (3CaO·Al_2_O_3_·CaCl_2_·10H_2_O) was found in both Paste-C and Paste-C70S30 under the long-term chloride ion erosion, whose characteristic diffraction peaks appeared around 11.2°, 22.6°, 23.4°, and 31.1° [51]. Meanwhile, the diffraction peaks of Friedel’s salt became stronger when 30% cement was replaced by slag powder, indicating that the chemical binding ability of paste to chloride ions was strengthened through supplying the C_3_A via slag powder [34]. As an important product of cement hydration, Ca(OH)_2_ showed characteristic diffraction peaks around 18°, 28.6°, and 34.1° in the XRD pattern. The Ca(OH)_2_ diffraction peaks in Paste-C70S30 were significantly weaker than those in Paste-C, which was consistent with the fact that consumption of Ca(OH)_2_ by slag powder was continuous through its pozzolanic reaction. The influence of polypropylene fibers on the hydration and erosion products was not investigated, because a conclusion had been made that fibers did not change the reaction products, according to the results in Figure 6.

### 3.3. Durability of CBRM Serving in the Subtidal Environment

As studied above, the effect of slag powder or polypropylene fibers on CBRM’s resistance against chloride ion erosion has been investigated, respectively. In Section 3.3, only Paste-C and Paste-C70S30P were included in the research, to explore the synergistic effect on the durability afforded by slag powder and polypropylene fibers. Furthermore, the environmental influence was discussed by the comparison between the intertidal environment and subtidal environment.

#### 3.3.1. Mechanical Properties

**Figure 17:**
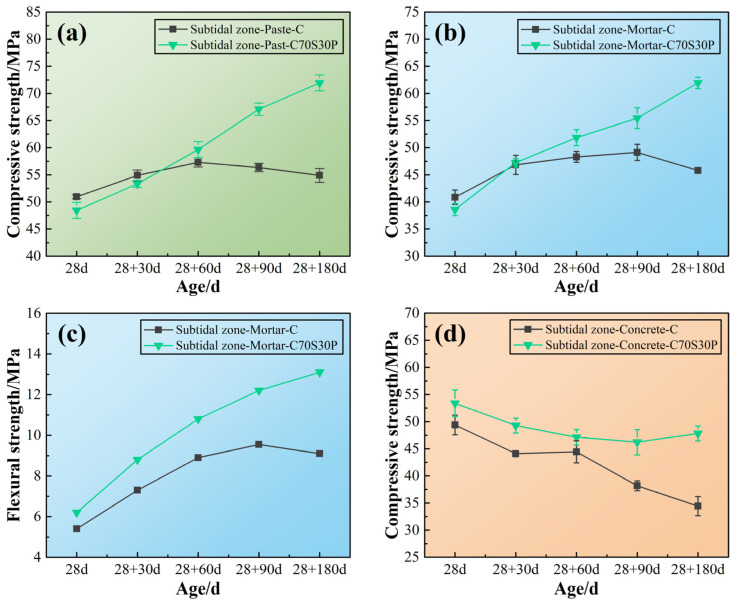
The mechanical properties of CBRM serving in the subtidal zone simulated environment. (**a**) CBRM-Paste, (**b**,**c**) CBRM-Mortar, and (**d**) CBRM-Concrete.

Figure 17 shows the mechanical properties of different types of CBRM in the completely immersed-coupled chloride ion erosion. Compared to subtidal mortar serving in the intertidal zone, the decay of the compressive or flexural strength in the subtidal zone was delayed from 60 to 90 days. After the 180-day simulated experiment, all CBRM, made by any cementitious materials or in any types, obtained better mechanical properties than those in the intertidal zone. The results show that the reinforcement effect of slag powder and polypropylene fibers was easier under the long-term immersion condition, because the water in the immersion solution played a role of water conservation, which completely inhibited the evaporation of water inside the CBRM and promoted the sequential hydration of cement and slag powder.

For CBRM-Paste and CBRM-Mortars without any additive, their mechanical properties increased first but then decreased. As for Mortar-C, its compressive strength decreased from 49.4 MPa at 28 days to 34.43 MPa at 28 + 180 days. No decay of mechanical properties of Paste-C70S30P and Mortar-C70S30P was found from the beginning to the end; on the contrary, their strength grew with the hydration of slag powder. With the addition of slag powder and polypropylene fibers, Paste-C70S30P had a compressive strength of 59.65 MPa at 28 + 60 days, higher than Paste-C, and kept the superiority ever since. A similar situation occurred with Mortar-C70S30P, but with Concrete-C70S30P, its strength decreased and then increased.

#### 3.3.2. Macroscopic Morphology of the Exposed Surface

**Figure 18:**
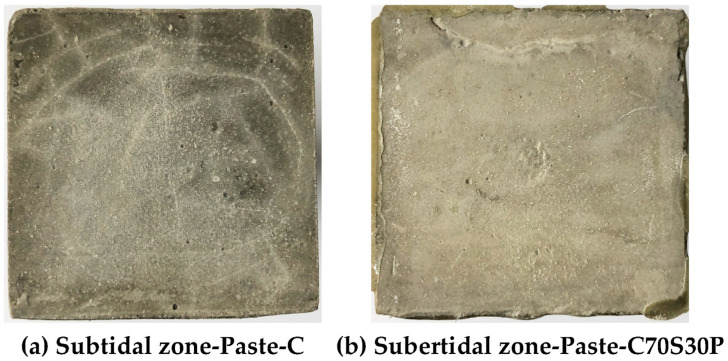
The synergistic influence of slag powder and polypropylene fibers on the macroscopic morphology of the exposed surface after 180 days of complete immersion-coupled chloride ion erosion.

Figure 18 shows the exposed surfaces of Paste-C and Paste-C70S30P that sustained complete immersion-coupled chloride ion erosion for 180 days. Compared with Figure 8, the deterioration of Paste-C in the subtidal zone is obviously better than that in the intertidal zone. The development trajectory of cracks on Paste-C was still dominated by arcs. No obvious fragments or debris were observed, which further proves that faced with the same chloride ion erosion, the complete immersion condition is gentler than the dry–wet cycles. However, the inconsistency of the surface color indicated that Paste-C had indeed been damaged. The exposed surface of Paste-C70S30P serving in the subtidal zone showed no cracks at all, except a very small amount of sodium chloride crystals adhered to the surface. The homogeneity of color on Paste-C70S30P’s exposed surface was evidently better than that on Paste-C, indicating the structural integrity of Paste-C70S30P. The above analysis again shows that slag powder and polypropylene fibers not only improved the mechanical properties of CBRM, but also enhanced its durability in the subtidal environment [52].

#### 3.3.3. Chloride Ions’ Penetration Depth

**Figure 19:**
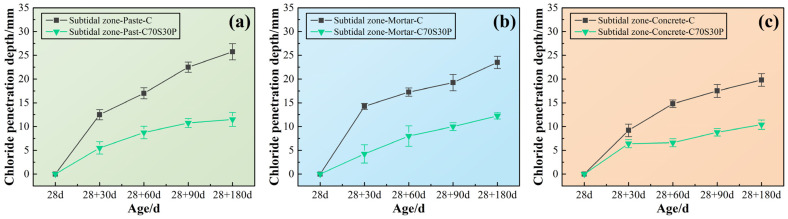
The chloride ions’ penetration depth of CBRM serving in the subtidal zone simulated environment. (**a**) CBRM-Paste, (**b**) CBRM-Mortar, and (**c**) CBRM-Concrete.

Figure 19 shows the effect of the simultaneous addition of slag powder and polypropylene fibers on the ability of CBRM in the subtidal zone to resist chloride ions’ penetration. The enhancement effect in the subtidal zone was more obvious with age, and the main reason may be that long-term immersion was beneficial to the hydration of slag powder, thereby increasing the ability unit volume to fix chloride ions. After 180 days of complete immersion erosion, the chloride ion erosion depths of Paste-C70S30P, Mortar-C70S30P, and Concrete-C70S30P were 11.5, 12.25, and 10.4 mm, respectively, which were only 44.66%, 52.13%, and 44.66% of Paste-C, Mortar-C, and Concrete-C. Furthermore, comparing the test results in the intertidal zone, it can be found that the chloride ions’ penetration ability was significantly weakened under the condition of complete immersion in the subtidal zone. Although the initial concentration of the immersion solution in the two environments was the same and updated regularly, the chloride ions’ penetration in the complete immersion environment only relied on the osmotic pressure, while in the dry–wet cycles environment, the chloride ions’ concentration gradient of the pore solution inside the material was changing, accelerating the penetration [5,27,53].

#### 3.3.4. Chloride Ions’ Profile

**Figure 20:**
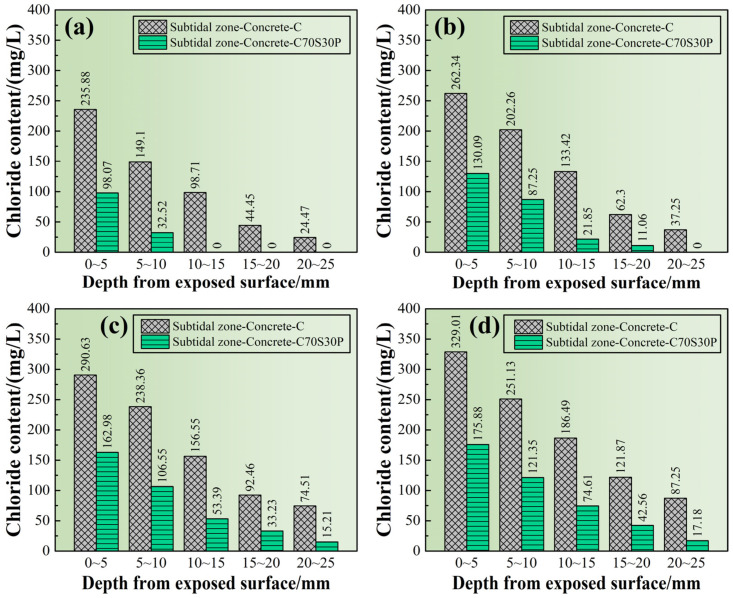
The chloride ions’ profile of CBRM-Concrete serving in the subtidal zone simulated environment. (**a**) 18 + 30 days, (**b**) 18 + 60 days, (**c**) 18 + 90 days, and (**d**) 18 + 180 days.

From the comparison in Figure 20, it was found that the slag powder and polypropylene fibers effectively reduced the chloride ions’ content in each region from the exposed surfaces. The presence of chloride ions in Paste-C70S30P was only detected in the 0~10 mm region after being immersed for 30 days, showing good corrosion resistance, while the chloride ions’ content in the region of 20–25 mm in Paste-C had reached 24.47 mg/L at 30 days. When the erosion progressed for 90 days, the chloride ions’ content in the region of 20–25 mm in Paste-C70S30P was detected at 15.21 mg/L for the first time. The chloride ions’ content of Paste-C in the 20–25 mm region increased from 74.51 mg/L at 90 days to 87.25 mg/L at 180 days. In contrast, the chloride ions’ content of Paste-C70S30P in the 20–25 mm region was detected to be 17.18 mg/L, indicating that the penetration rate of chloride ions in it had been very slow since 90 days. It can be seen that slag powder and polypropylene fibers help cement to improve the resistance to chloride ions’ penetration [18,27].

## 4. Conclusions

(1) The durability of the same CBRM in the supratidal zone, intertidal zone, and subtidal zone was significantly different. From gentle to severe, the bank protection environments can be ordered as supratidal zone > subtidal zone > intertidal zone. The high-humidity air in the supratidal zone was conducive to CBRM’s mechanical properties, but the chloride ions in the subtidal water invaded the CBRM slowly, causing surface cracking. The dry–wet cycles in the intertidal zone accelerated the chloride ion erosion, leading to the more rapid deterioration of mechanical properties.

(2) Using CBRM prepared only with cement as a cementitious material made it difficult to resist chloride ion erosion in the intertidal or subtidal zone. The chloride ions penetrating into the materials promoted the formation of Ettringite in the internal pores, gradually resulting in local cracking. Non-hydrated cement particles wrapped by hydration products were exposed after long-term erosion, and their loose structure further accelerated the penetration of chloride ions.

(3) Slag powder upgraded the chloride ions’ resistance of CBRM by improving the hydration products of cement, increasing its physical adsorption ability and chemical binding ability to chloride ions. The pozzolanic reaction of slag powder promoted the structure of C-S-H gels to be more amorphous, providing more sites for adsorption of chloride ions. The hydration of C_3_A (supplied by slag powder) and chloride ions resulted in Friedel’s salt, which hindered the further migration of chloride ions.

(4) Polypropylene fibers did not change the type of hydration products, but their hydrophobicity effectively delayed the penetration of immersion solution and improved the erosion resistance of CBRM. Tensile stress is generated by fibers to avoid parts of the matrix being separated from each other after the cracking of CBRM, which makes up for the defects of high brittleness and low toughness of CBRM, and finally controls the number and the shape of cracks.

(5) Using 30 wt.% slag powder instead of cement and synergistically adding 0.1 vol.% of polypropylene fibers can realize the complementary advantages of these two additives in chloride ion erosion resistance. The structural optimization by polypropylene fibers increased the strength of CBRM at 28 days, showing good durability for the short term, and the composition improvement by slag powder enhanced the compactness of the hardened cement matrix, fundamentally ensuring good durability for the long term.

## Figures and Tables

**Table 1:** The chemical compositions of cement and slag powder (wt.%).

Oxides	SiO_2_	Al_2_O_3_	Fe_2_O_3_	CaO	MgO	SO_3_	R_2_O	LOI
Cement	22.73	4.34	2.75	62.96	1.81	2.55	0.59	2.27
Slag powder	38.54	6.87	0.95	43.44	6.55	0.70	0.70	2.25

**Table 2:** The mix ratio of CBRM-Paste.

Specimens	C	S	P (vol. %)	W/B Ratio
Paste-C	100%	0	0	0.43
Paste-C70S30	70%	30%	0	0.43
Paste-CP	100%	0	0.10%	0.43
Paste-C70S30P	70%	30%	0.10%	0.43

**Table 3:** The mix ratio of CBRM-Mortar.

Specimens	C	S	Sand	P (vol. %)	W/B Ratio
Mortar-C	100%	0	300%	0	0.43
Mortar-C70S30	70%	30%	300%	0	0.43
Mortar-CP	100%	0	300%	0.10%	0.43
Mortar-C70S30P	70%	30%	300%	0.10%	0.43

**Table 4:** The mix ratio of CBRM-Concrete (kg/m^3^).

Specimens	C	S	Sand	Stone	P (vol. %)	PCE	W/B Ratio
Concrete-C	384	0	836	1065	0	1.92	0.43
Concrete-C70S30	268.8	115.2	836	1065	0	1.92	0.43
Concrete-CP	384	0	836	1065	0.91	1.92	0.43
Concrete-C70S30P	268.8	115.2	836	1065	0.91	1.92	0.43

## Data Availability

Not applicable.
